# Targeted Polymer–Peptide Conjugates for E-Selectin Blockade in Renal Injury

**DOI:** 10.3390/pharmaceutics17010082

**Published:** 2025-01-09

**Authors:** Nenad Milošević, Marie Rütter, Yvonne Ventura, Valeria Feinshtein, Ayelet David

**Affiliations:** Department of Clinical Biochemistry and Pharmacology, Faculty of Health Sciences, Ben-Gurion University of the Negev, Beer-Sheva 84105, Israel

**Keywords:** cell adhesion molecules, E-selectin, VCAM-1, P-selectin, “drug-free” macromolecular therapeutics, HPMA, kidney inflammation, nanomedicines

## Abstract

Background/Objectives: Leukocytes play a significant role in both acute kidney injury (AKI) and chronic kidney disease (CKD), contributing to pathogenesis and tissue damage. The process of leukocyte infiltration into the inflamed tissues is mediated by the interactions between the leukocytes and cell adhesion molecules (CAMs, i.e., E-selectin, P-selectin, and VCAM-1) present on the inner surface of the inflamed vasculature. Directly interfering with these interactions is a viable strategy to limit the extent of excessive inflammation; however, several small-molecule drug candidates failed during clinical translation. We hypothesized that a synthetic polymer presenting multiple copies of the high-affinity E-selecting binding peptide (P-Esbp) could block E-selectin-mediated functions and decrease leukocytes infiltration, thus reducing the extent of inflammatory kidney injury. Methods: P-Esbp was synthesized by conjugating E-selecting binding peptide (Esbp) to N-(2-hydroxypropyl)methacrylamide (HPMA) copolymer with reactive ester groups via aminolysis. The effects of P-Esbp treatment on kidney injury were investigated in two different models: AKI model (renal ischemia—reperfusion injury—RIRI) and CKD model (adenine-induced kidney injury). Results: We found that the mRNA levels of E-selectin were up-regulated in the kidney following acute and chronic tissue injury. P-Esbp demonstrated an extended half-life time in the bloodstream, and the polymer accumulated significantly in the liver, lungs, and kidneys within 4 h post injection. Treatment with P-Esbp suppressed the up-regulation of E-selectin in mice with RIRI and attenuated the inflammatory process. In the adenine-induced CKD model, the use of the E-selectin blocking copolymer had little impact on the progression of kidney injury, owing to the compensating function of P-selectin and VCAM-1. Conclusion: Our findings provide valuable insights into the interconnection between CAMs and compensatory mechanisms in controlling leukocyte migration in AKI and CKD. The combination of multiple CAM blockers, given simultaneously, may provide protective effects for preventing excessive leukocyte infiltration and control renal injury.

## 1. Introduction

The development of effective pharmacological treatments for acute kidney injury (AKI) and chronic kidney disease (CKD) remains a significant challenge in global health [1]. Despite ongoing research efforts, there are currently no approved drugs specifically designed to prevent, treat, or enhance recovery from kidney injury, and the current strategies primarily focus on preventing the further deterioration of renal function [2]. Leukocytes play an important role in renal inflammation, contributing to both harmful and protective effects [3]. Leukocyte trafficking into the inflamed tissue is a crucial component of the inflammatory response; however, when dysregulated or excessive, it can further contribute to pathological development [4,5,6]. The entry of leukocytes into inflamed tissues is regulated by their interactions with endothelial cells, specifically with the cell adhesion molecules (CAMs) expressed on the endothelium surface. The major endothelial-expressed CAMs can be divided into integrins, selectins (E-, L-, and P-selectin), and immunoglobulin superfamily members (ICAM-1 and VCAM-1), each of them mediating different stages of leukocyte trafficking [7]. Due to their role, CAMs have been recognized as viable targets for reducing leukocyte influx and inhibiting excessive inflammation [6,8] with the aim of reducing tissue injury. Several CAM inhibitors have been employed so far in preclinical development, including recombinant ligands [8], mAbs against selectins [9,10] and small molecular inhibitors (glycomimetics) [11,12]. However, their clinical translation has stagnated due to a combination of factors including insufficient binding affinity (glycomimetics), unfavorable pharmacokinetics (PK), or, in most cases, due to a lack of efficacy.

Some of these challenges could be overcome by designing CAM-targeted nanomedicines decorated with multiple high-affinity ligands for increased binding avidity to CAMs. Due to their large size, targeted nanomedicines (such as nanoparticles, liposomes, and polymer–drug conjugates) exhibit significantly longer half-life times in circulation compared to free small-molecule drugs [13]. Although such nanomedicines are usually intended as drug delivery platforms, a more recent approach aims to develop systems that exert biological activity without the need to add low-molecular-weight drugs. These systems, termed drug-free macromolecular therapeutics (DFMTs), exert their effects through strong binding to their intended targets and blocking their function [14,15].

We previously reported the synthesis of an HPMA-based polymer bearing multiple copies of the high-affinity E-selectin binding peptide (Esbp; primary sequence DITWDQLWDLMK) intended to target E-selectin on activated endothelium. This polymer (designated P-Esbp) was, at first, utilized as a drug delivery platform for a cytotoxic drug (doxorubicin) payload to the tumor vasculature [16,17,18]. The “drug-free” version of the polymer, without any drug attached, was shown to inhibit the metastatic spread of melanoma by blocking E-selectin (in this case, the E-selectin blockade interfered with the attachment of circulating cancer cells to the inflamed endothelium) [17]. Moreover, P-Esbp was shown to inhibit leukocyte recruitment to inflamed vasculature and reduce atherosclerotic lesion development in atherosclerotic mice [19] and was further demonstrated to attenuate neutrophil-mediated liver injury in a mice model of alcohol-related liver disease [20].

In this work, we investigated the ability of P-Esbp to attenuate kidney inflammation in mice models. Kidney inflammation was chosen as P-Esbp accumulates at high concentration in the kidneys of mice (in addition to liver tissue) [20]. The renal ischemia–reperfusion injury model (RIRI) is a surgical model representing a more acute type of kidney inflammation, with pronounced neutrophil infiltration that is mediated, in part, by E-selectin [21]. The second kidney inflammation model that we chose mimics several aspects of CKD and is induced by feeding mice food that is high in adenine [22]. The consumption of adenine-enriched food leads to the formation of an adenine metabolite—2,8-dihydroxyadenine—which forms crystals within renal tubules and induces renal injury and inflammation, followed by the loss of kidney function [23].

We tested whether the E-selectin blockade by P-Esbp could inhibit leukocyte infiltration into the inflamed kidneys, restrict inflammation, and exert beneficial effects on overall kidney function in these mice models.

## 2. Materials and Methods

### 2.1. Chemical Synthesis and Characterization of Polymers P-Esbp

All chemicals were of reagent grade and were obtained from Sigma-Aldrich (Rehovot, Israel) unless stated otherwise. The N-terminal Lysine-harboring E-selectin-binding peptide (Esbp, KDITWDQLWDLMR) and the control peptide with a scrambled Esbp sequence (EsbpScrm, KRMIDWTWLQLDD) were purchased from GL Biochem Ltd. (Shanghai, China). HPMA monomer was purchased from Polysciences (Warrington, PA, USA). The monomers methacryloyl-glycylglycine p-nitrophenyl ester (MA-GG-ONp) and methacryloyl-aminopropyl fluorescein-5-isothiocyanate (MAP–FITC) were synthesized as described previously [16]. 

P-Esbp was synthesized by coupling the N-terminal lysine-harboring Esbp or EsbpScrm to an HPMA-based precursor copolymer with reactive ester (O-nirtophenyl—ONp) groups (P-(GG-ONp)-FITC) via ONp aminolysis, as described previously [17]. The conjugates were isolated and purified on a PD-10 column using double-distilled water as the eluent. The content of conjugated peptides was estimated via ^1^H-NMR, at 500 Hz, using the Tryptophan (Typ, W) proton chemical shift (δ 6.9–7.6, m, 10 H for Esbp/EsbpScrm). P-Esbp-IR783 was synthesized as described previously [17].

### 2.2. Pharmacokinetic and Biodistribution Analysis of P-(Esbp)-IR783

The PK parameters of P-Esbp were analyzed in healthy BABL/c mice to provide a baseline for understanding how the polymer distributes throughout the body without the influence of kidney injury. P-(Esbp)-IR783 (Mw: 46.7 kDa; P_I_: 1.19; 3 mol% Esbp; 5 mol% IR783) was administered to 8-week-old female BALB/c mice (1 mg polymer/mice; n = 3) via tail vein injection. Animals were euthanized at designated time points (1 min, 5 min, 15 min, 30 min, 1 h, 2 h, 4 h, 8 h, 24 h, and 48 h). Blood and other major organs/tissues including hearts, lungs, kidneys, livers, and spleens were isolated following euthanasia. Serum sample fluorescent intensity was measured using the Infinite M-200 microplate fluorescence reader (Tecan, Männedorf, Switzerland). Corresponding serum concentrations were calculated using a previously constructed calibration curve prepared with known concentrations of the same polymer in human plasma. Major organs were imaged individually, and regions of interest (ROIs) were analyzed using the IVIS-Lumina imaging system without perfusion. The PK parameters, such as total clearance (CL), volume of distribution (Vd), and biological half-life (t_1/2_), were determined using the bolus intravenous input non-compartmental and two-compartmental analysis of WinNonlin. The area under the curve (AUC) was calculated using the trapezoidal rule. 

### 2.3. Animal Models of Kidney Inflammation

All animal experiments were approved and performed in compliance with the standards of the Ben-Gurion University of the Negev (BGU) Institutional Animal Care and Use Committee (IACUC), protocol code IL-56–08-2019 (C), period of authorization from 09/12/2019 through 09/11/2022. Male C57BL/6 mice and female BALB/c mice were obtained from Harlan Biotech Israel (HBI), (Rehovot, Israel) and housed in the animal facility of the Ben-Gurion University of the Negev.

#### 2.3.1. In Vivo Model of Acute Kidney Inflammation—Renal Ischemia–Reperfusion Injury Model (RIRI)

The surgical procedure was based on the adapted protocol from a publication by Singbartl and Ley [24,25]. During initial experiments, high mortality was observed with 32 min bilateral RIRI. In an attempt to reduce the mortality, 25 min of bilateral RIRI was performed in the following experiments. According to Wei et al. [25], the rise in the blood urea nitrogen (BUN) levels is similar with both 25 and 30 min ischemia. 

C57BL/6 mice (age: 8–10 weeks; male; bodyweight more than 20 g) were anesthetized with i.p. injection of ketamine and xylazine (100 mg/kg, 10 mg/kg). After confirming the depth of surgical anesthesia, mice were shaved, and the operating area was disinfected. Mice body temperature was measured using the thermostatic station with a rectal probe, and mice were left to stabilize their body temperature to 37 ℃. During the surgery, body temperature was monitored and kept at 37 ± 0.4 ℃, as this is crucial for the reproducible kidney injury [26]. Mice skin and muscles were cut to expose renal pedicle first on the left and subsequently on the right side, as shown in Supplementary Figure S1A. The left renal artery was clamped with a Micro Serrefines clamp (Fine Scientific Tools—Supplementary Figure S1B); after confirmation of the color change in the kidney tissue following blood occlusion, the muscle layer was closed with one suture. The right renal artery was also clamped (the time difference between left and right clamping was less than 2 min) and the skin was closed with one suture. Following 25 min of ischemia time, the sutures were opened, the Micro Serrefines clips were removed, and the kidneys were inspected for color change (from dark red to a physiological, light brown color), which is an indicator of successful reperfusion. After closing the muscle layer and skin, mice received 1 mL of heated saline and Buprenorphine (dose: 50 µg/kg subcutaneously). Mice were randomized into groups, as follows: A—only RIRI; B—RIRI and i.p. 1 mg of P-Esbp in saline after reperfusion and another dose of 1 mg of P-Esbp in saline the following morning; and C—sham-operated mice (group C underwent all the procedures as A except for the clamping of the renal arteries). Animals were given thermal support by IR lamps and were followed until regaining full consciousness. All the procedures and time points were recorded for each mouse to exclude any mice where some deviations from the protocol occurred (using surgery logbook—Supplementary Figure S1C). Mice were euthanized exactly 24 h after reperfusion time. Upon euthanasia, blood samples and kidney tissue samples were collected for biochemical analysis and tissue mRNA expression via qRT-PCR, as described below.

#### 2.3.2. In Vivo Model of Adenine-Induced Chronic Kidney Disease (CKD)

Experimental Design of Adenine-Induced CKD Murine Model

A casein-based diet with adenine (TD.130900–Adenine Diet (0.2% adenine, total phosphate content 0.9%, and total calcium content 0.6%), purchased from Harlan Biotech Israel (HBI), Rehovot, Israel) was formulated to have similarities to the diet described by Jia et al. [22]. The vitamin mix was increased by 50% and 2 ppm additional vitamin K and 10 ppm additional thiamin HCl were added to make the diet more suitable for irradiation sterilization. The control diet was also purchased from the same vendor and was identical in formulation to the adenine diet, apart from lacking adenine. 

For the calibration experiments, 8-week-old male C57BL/6 mice were randomized into groups receiving either the adenine diet 0.2% for 5 days (n = 4), 14 days (n = 3), and 25 days (n = 6) or the control diet (n = 9; euthanized on days 14 and 25). Three mice were housed per cage. Every second day, mice body weight (BW) was determined as well as average food intake. On the designated days, mice were euthanized, and serum and kidney tissue samples were obtained and processed for further biochemical, histological, and mRNA expression analyses. 

In the interventional experiment, C57BL/6 male mice were randomized into groups receiving the 0.2% adenine diet for 25 days and i.p. injections of either P-Esbp, P-EsbpScrm, or saline; the control group received the control diet and saline injections. Treatments were administered every second day from day 6 in the form of 1 mg of the corresponding polymer dissolved in 200 µL of saline.

Tissue and Serum Processing and Analysis

Total RNA was extracted from kidney samples with TRIzol reagent and was transcribed into cDNA using a High-Capacity cDNA Reverse Transcription Kit. RT-PCR was performed using Taqman probes for E-selectin, TNF-α, and IL-1β, which were normalized to the expression levels of GAPDH (Supplementary Table S1). The results are expressed as fold induction relative to the control-fed groups. Upon blood collection, full blood was let to clot for 15–30 min at room temperature. Following centrifugation at 2000× *g* for 10 min, serum was collected and kept at 4 °C. Serum creatinine and serum urea levels were determined using a clinical chemistry analyzer Beckman Coulter AU5800 (Soroka Medical Center, Beer Sheva, Israel).

## 3. Results

### 3.1. Targeted Polymer Synthesis

**Figure 1:**
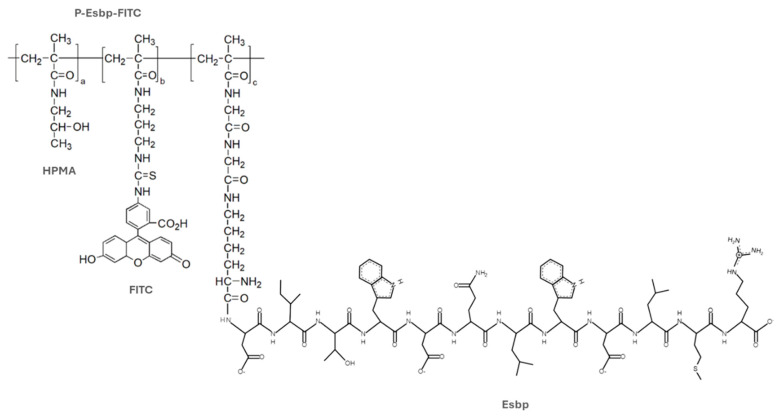
Structure of the FITC-labeled E-selectin-binding polymer (P-(Esbp)-FITC). HPMA indicates N–(2–hydroxypropyl)methacrylamide; Esbp—E-selectin-binding peptide. FITC—fluorescein isothiocyanate.

A polymer precursor for Esbp conjugation (P-(GGONp)-FITC) was synthesized, purified, and characterized as previously reported [16,17,20]. The molecular weight of P-(GGONp)-FITC was 34 kDa (determined via size-exclusion chromatography on an ACTA-FPLC), which is below the renal glomerular filtration threshold, allowing for the polymer to be renally excreted. The content of the reactive group for peptide conjugation—ONp—was 7.5 mol% (determined spectrophotometrically). P-Esbp was synthesized and characterized as previously described [20]. The structure of the FITC-labeled, E-selectin-binding polymer (P-(Esbp)-FITC) is shown in Figure 1. The characteristics of the synthesized polymer and control (P-(EsbpScrm)-FITC) are shown in Table 1. The content of conjugated peptides was estimated via NMR using the ratio of signal intensities of tryptophan present in the peptide sequence (δ 6.9–7.6, m, 10H) and the signal of HPMA (δ 0.9–1.1, m, 6H). The content of Esbp/EsbpScrm in P-Esbp/P-EsbpScrm was estimated close to the theoretical maximum (the content of ONp groups), indicating the full conversion of reactive ONp groups (Table 1).

### 3.2. Pharmacokinetics (PK) and Biodistribution (BD) Analysis of P-(Esbp)-IR783

**Figure 2:**
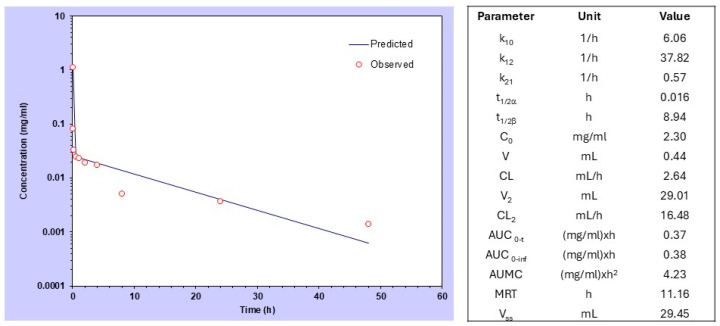
Compartmental analysis and pharmacokinetic data of P-(Esbp)-IR783 in healthy mice after intravenous bolus injection.

We first analyzed the PK and BD characteristics of P-Esbp in healthy BALB/c female 8-week-old mice using near-infrared (NIR) optical imaging. The half-life time of HPMA-based copolymers in circulation can vary significantly, depending on the molecular weight and the specific composition of the polymer [27]. P-Esbp-IR783 (Mw~47 kDa) has a distribution (*t*_1/2_ alpha) of approximately 1 minute and an elimination half-life (*t*_1/2_ beta) of 8.94 h. The blood data were fitted with a two-compartmental model (Figure 2), consistent with other examples of HPMA-based copolymer conjugates. 

**Figure 3:**
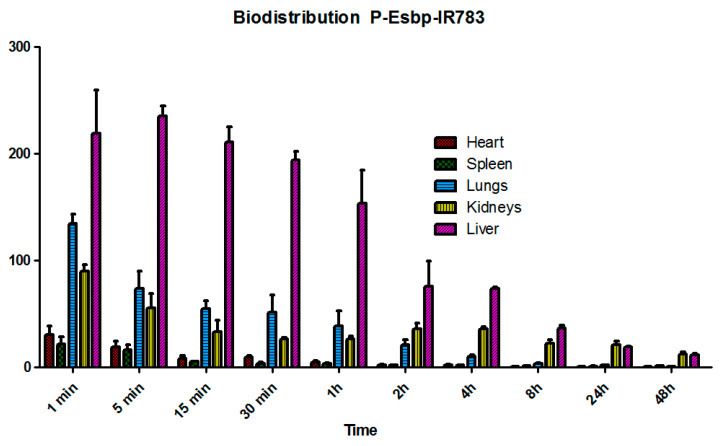
The biodistribution profile of P-Esbp-IR783 in heathy mice.

P-(Esbp)-IR783 accumulated significantly in the liver of healthy mice, and a substantial amount of polymer was also detected at the first 4 h in the lungs and kidneys (Figure 3). Detectable levels were observed in the liver and kidneys even 48 hours post-injection. The liver, lungs, and kidneys are characterized by high tissue perfusion and discontinuous vascular walls (these fenestrae are generally between 50 and 100 nanometers in diameter) that allow substances circulating in the plasma to extravasate. These results align with other examples of polymer–drug conjugates or nano-sized formulations, such as liposomes, polymeric micelles, and nanoparticles [28]. The significant perfusion and accumulation of P-Esbp in the kidneys highlight its potential use for treating kidney diseases by targeting the E-selectin that is present at the luminal aspect of inflamed blood vessels. Since E-selectin expression levels are significantly up-regulated in response to inflammatory stimuli, P-Esbp may substantially accumulate in the blood vessels of the injured kidney and the renal localization is expected to be higher.

### 3.3. P-Esbp Reduced Kidney Damage in Acute Kidney Inflammation—Renal Ischemia–Reperfusion Injury (RIRI) Model

Renal ischemia–reperfusion injury (RIRI) is a model of acute renal damage, and some reports have suggested the important role of E-selectin in mediating the inflammatory response following reperfusion [24]. In a series of experiments, we investigated the effects of P-Esbp on renal injury parameters and the renal expression of E-selectin to determine if it can influence AKI. 

**Figure 4:**
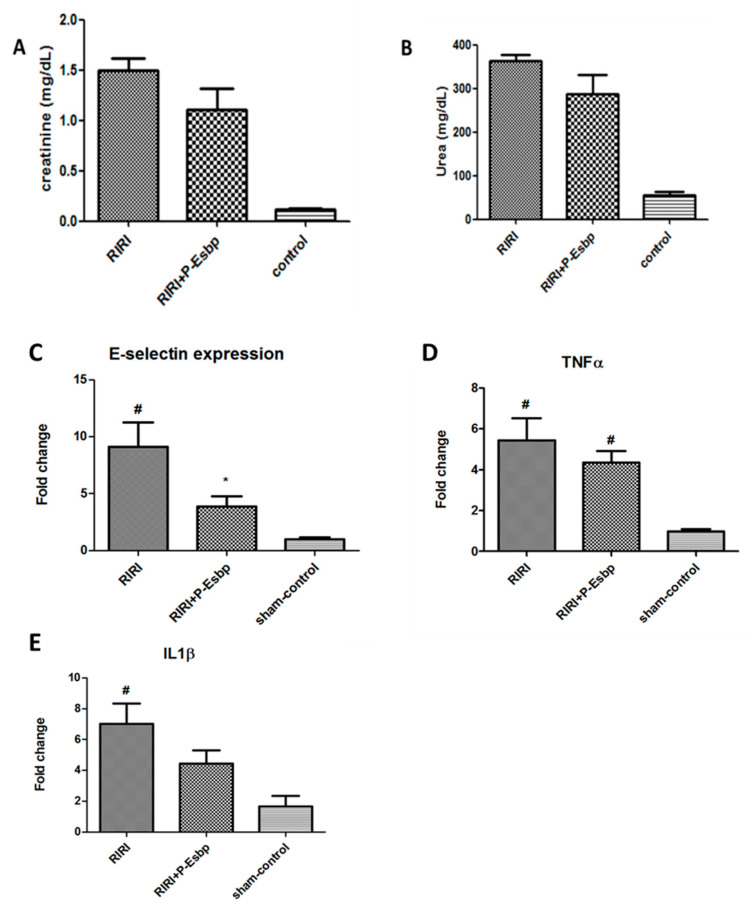
Effects of P-Esbp treatment (1 mg in 200 µL of saline at the time of reperfusion and 1 mg the following morning) on kidney function biochemical parameters—creatinine (**A**) and urea (**B**) 24 h after 25 min RIRI. Renal tissue expression levels of E-selectin (**C**), TNFα (**D**), and IL-1β (**E**) 24 h after 25 min RIRI. #—statistically significant difference compared to the sham-control group, *p* < 0.05; *—statistically significant difference compared to the RIRI group, *p* < 0.05.

In this experimental setting, kidney injury parameters (i.e., urea and creatinine) were elevated after the procedure compared to sham-operated mice. P-Esbp treatment reduced the level of kidney damage, in accordance with Singbartl [24], yet the effects were not statistically significant (Figure 4A,B). Further analysis of renal tissue expression of E-selectin revealed that E-selectin mRNA levels were up-regulated by about 8-fold in the kidney of RIRI mice relative to sham-control mice. This marked up-regulation of E-selectin is crucial in initiating the inflammatory cascade and plays a pivotal role in facilitating neutrophil recruitment to the injured tissue. P-Esbp treatment significantly suppressed the up-regulation of E-selectin in RIRI mice (Figure 4C). The expression levels of pro-inflammatory cytokines—TNFα and IL-1β (Figure 4D,E)—were lower in the P-Esbp-treated group relative to untreated RIRI group, indicating that P-Esbp therapy attenuates inflammatory processes in the acute experimental setting of renal injury.

### 3.4. Continuous P-Esbp Treatment Did Not Affect Chronic Kidney Injury in Adenine-Induced CKD Model

Previous studies indicated the development of chronic kidney injury in mice fed with an adenine-rich 0.2% diet [29,30,31]. In the first experiment, we investigated the effects of an adenine-rich diet on biochemical, and inflammatory cytokine parameters, especially on the expression patterns of CAMs in renal tissues on days 5, 14, and 25 after starting the diet.

**Figure 5:**
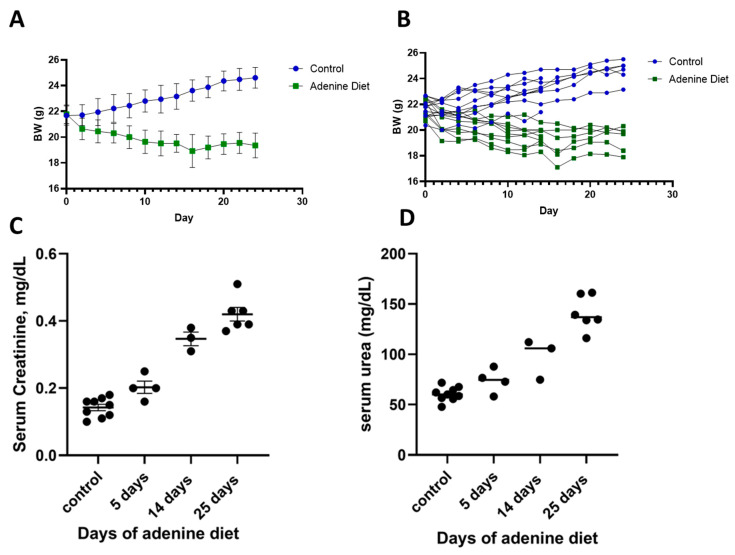
Induction of CKD by adenine diet (0.2%). Mice body weight as group average (**A**) and individual weight (**B**). Biochemical parameters of serum creatinine (**C**) and serum urea (**D**) at different time points from the initiation of the adenine diet.

Adenine-fed C57BL/6 mice experienced a reduction in BW, which was more pronounced in the first week of feeding and stabilized in the following weeks (Figure 5A,B—individual profiles). This reduction in BW was comparable with that reported in the literature, and, on average, it was around 10% of the original weight [29,32]. The average food intake was 3.2 g of food per mouse per day in the control group and 2.2 g per mouse per day in the adenine group. Kidney injury was confirmed by elevated serum levels of creatinine and serum urea (Figure 5C,D) and their levels gradually increased throughout the course of the experiment. 

**Figure 6:**
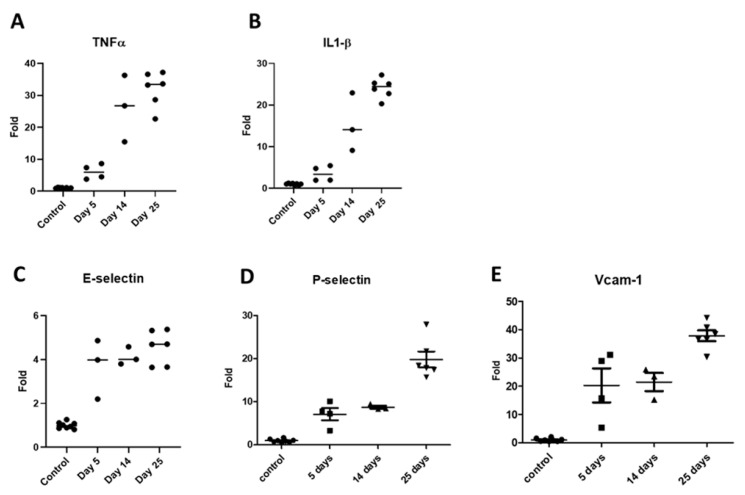
mRNA expression patterns of inflammatory markers in renal tissues of adenine-rich diet-fed mice. TNFα (**A**); IL-1β (**B**); E-selectin (**C**); P-selectin (**D**); and VCAM-1 (**E**).

The inflammatory cytokines TNFα and IL-1β also showed a rising trend in mRNA expression (Figure 6A,B), with a gradual increase in their levels from day 5 to day 25. Endothelial CAMs are overexpressed in response to inflammation and signaling by cytokines. Our results showed that the CAMs E-selectin, VCAM-1, and P-selectin were up-regulated in kidney tissues of adenine-fed mice. The E-selectin levels were elevated at the earliest time point of 5 days and this trend continued for time points at 14 days and 25 days (Figure 6C), demonstrating the highest folds at the last time point (4.6-fold average). Both P-selectin (Figure 6D) and VCAM-1 (Figure 6E) expression levels were enhanced during the course of the experiment, and the fold increase in their mRNA levels was about 5–10-times higher than that of E-selectin. 

**Figure 7:**
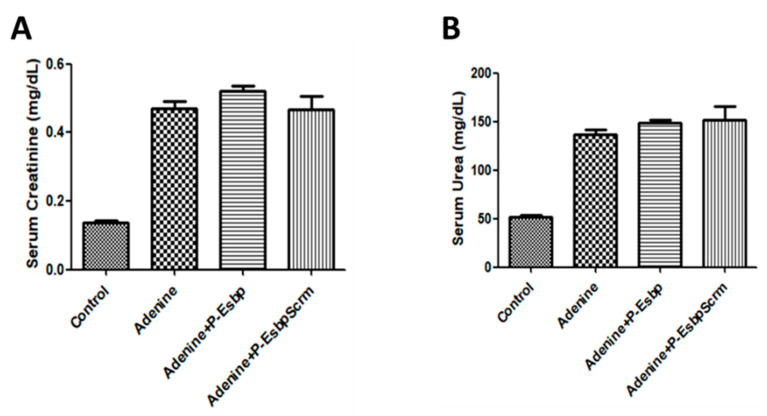
Effects of P-Esbp treatment (1 mg in 200 µL of saline i.p. every second day from day 6 of the experiment; total of ten doses) on kidney function biochemical parameters from the interventional experiment. Serum creatinine (**A**) and serum urea (**B**) upon animal euthanasia on day 28.

To investigate the effects of E-selectin blockage with P-Esbp, adenine-fed mice received 10 i.p. injections of P-Esbp or the polymer with the scrambled version of the peptide—P-EsbpScrm. Since the half-life time of P-Esbp is approximately 9 h in circulation, and it takes about 4 to 5 half-lives for an almost complete clearance from the body, P-Esbp was injected once every two days to ensure a continuous dose of the polymer conjugate in circulation, and to ensure that it was available for endothelial E-selectin blockade under chronic conditions. In line with previously described results, biochemistry parameters, serum creatinine, and serum urea (Figure 7A,B) were profoundly higher in mice fed with an adenine diet throughout the experiment (from day 5 to day 25). However, treatment with P-Esbp did not inhibit the rise in serum creatinine and urea levels. The lack of therapeutic efficacy may be attributed to the complementary roles of CAMs. The level of P-selectin was significantly up-regulated in adenine-fed mice. E- and P-selectin function cooperatively and can compensate each other in various biological processes. We thus assume that targeting and blocking all the three CAMs (E-selectin, P-selectin, and VCAM-1), simultaneously, might provide therapeutic benefits. A suboptimal dosing regimen of P-Esbp can also explain the results. Overall, in the chosen animal model, feeding protocol, and dosing regimen, E-selectin blockade cannot substantially inhibit chronic kidney injury and inflammation caused by adenine diet. 

## 4. Discussion

In this study, we investigated different models of kidney inflammation and identified those with a clear involvement of CAMs. In the RIRI model of rapid and acute inflammation, E-selectin mRNA was about eight times higher 24 after ischemia and reperfusion, indicating that E-selectin plays a significant role in the early inflammatory response following AKI. Treatment with P-Esbp attenuated inflammatory processes in RIRI mice by significantly suppressing the up-regulation of E-selectin expression. This is in line with previous studies showing that the blockade of E-selectin or P-selectin (by a monoclonal antibody or small-molecule selectin ligand) decreases neutrophil recruitment into the kidney and preserves organ morphology and function and in sepsis-induced AKI [33,34,35]. Yet, polymer treatment only mildly influenced kidney injury parameters (i.e., urea and creatinine). In a model of more gradual, chronic kidney inflammation, we confirmed the up-regulation of all three CAMs (E-selectin, P-selectin, and VCAM-1) in kidney samples from adenine-fed animals. While E-selectin expression was up-regulated approximately 8-fold in RIRI after 24 h, its levels increased only 2-fold in CKD at the initial time point (day 5) and reached 4–5-fold on day 25. P-selectin and VCAM-1 have reached higher folds of up-regulation compared to E-selectin (8-fold and 20-fold, respectively, on day 5, and 20–35 fold of increase on day 25). This indicates that P-selectin and VCAM-1 play a more substantial role in the progression of CKD than E-selectin. Multiple i.p. injections of P-Esbp in the adenine-diet-induced CKD model did not protect mice from kidney injury. The physiological role of P-selectin is to work synergistically with E-selectin in the mediation of initial leukocyte adhesion to activated endothelium during acute and chronic inflammation. It is possible that due to the overlapping and mutually compensating functions of selectins, the blockade of only one selectin family member was not sufficient for inhibiting chronic inflammation and renal injury. For comparison, in an inflammatory model of alcohol-induced liver injury (the NIAAA model), where E-selectin is the sole CAM that was up-regulated to a significant extent [20,36], E-selectin blockade by P-Esbp showed a profound anti-inflammatory efficacy. The increase in P-selectin and VCAM-1 expression in RIRI was less pronounced after 24 h when compared to CKD after 5 days. Specifically, the upregulation was approximately six times higher for P-selectin and twelve times higher for VCAM-1 in RIRI [37,38,39], which is ~ three times lower relative to their up-regulation in CKD. This might explain the beneficial effects of P-Esbp observed in RIRI but not in CKD. Future experiments with the combination of E-selectin, P-selectin, and VCAM-1 blockers given simultaneously might attenuate the manifestation of adenine-diet-induced kidney injury. Our results show that therapeutic success in treating one disease is not a guarantee for benefit across different pathologies. This is evident in several other drug candidates (i.e., Inclacumab, a monoclonal antibody against P-selectin, was dropped for cardiovascular diseases treatment and is now in trials for the treatment of sickle cell disease [40]). Effectively blocking a single CAM might be beneficial in one inflammatory setting, but might provide limited efficacy in others [41,42].

Taken together, the results from this study give several insights on the process of developing a polymer–peptide conjugate specifically designed to target and block E-selectin to prevent renal injury. E-selectin has a more significant and immediate role in the inflammatory processes in acute compared to chronic renal injury. The upregulation of E-selectin in response to inflammatory stimuli was more pronounced in AKI than CKD. Treatment with P-Esbp suppressed the up-regulation of E-selectin in mice with AKI. The mild protective effects in the models of kidney inflammation highlight the interconnected nature of CAMs and their different individual contributions to the specific pathological process. Careful monitoring of potential compensatory increases in other CAMs is crucial when targeting E-selectin. Overall, more effort should be invested in precisely characterizing different inflammatory diseases and/or animal models to pinpoint those where the blockage of one or several CAMs would provide the most therapeutic benefits. 

## Figures and Tables

**Table 1:** Characteristics of synthesized polymers and precursor copolymers.

HPMA Copolymer	Mw [kDa] ^a^	Polydispersity ^b^	%mol FITC or IR783 ^c^	%mol ONp/Peptide/Scrm ^d^
P-(GGONp)-FITC	34.0	1.42	1.8	7.50
P-(Esbp)-FITC	34.1	1.35	1.8	7.49
P-(EsbpScrm)-FITC	33.2	1.2	1.8	7.35

^a,b^ Weight-average molecular weight (Mw) and polydispersity (PI) of precursors and copolymer peptide conjugates were estimated via size-exclusion chromatography on an ACTA-FPLC system, using a Sephacryl 16/60 S-400 column (GE Healthcare) calibrated with fractions of known-molecular-weight HPMA homopolymers or by using the GPC/ HPLC Shimadzu system equipped with UV-VIS, refractive index, and multiangle light scattering DAWN 8 EOS (Wyatt Technology Corp., Santa Barbara, CA) detectors using a TSK 3000 SWXL column (Tosoh Bioscience, Japan). ^c^ The contents of FITC residues were determined by measuring the UV absorbance at 492 nm (ε = 82,000 M^−1^ cm^−1^). ^d^ The contents of peptide-targeting moieties were estimated via ^1^H-NMR at 500 Hz using the Tryptophan (Typ, W) chemical shift of aromatic amino acids (δ 6.9–7.6, m, 10H).

## Data Availability

Data will be made available upon reasonable request.

## Supplementary Materials

The following supporting information can be downloaded at: https://www.mdpi.com/article/10.3390/pharmaceutics17010082/s1, Figure S1: The incision locations for left and right kidney clamping-adapted from Ref. [25]; Table S1: Primer sequences for real-time PCR.
