# Supplementary material for: Targeted Polymer–Peptide Conjugates for E-Selectin Blockade in Renal Injury
Source: Pharmaceutics. 2025 Jan 9;17(1):82. doi: 10.3390/pharmaceutics17010082 (PMC11768228; doi:10.3390/pharmaceutics17010082)
Supplement: Supplementary file 1 [file pharmaceutics-17-00082-s001.zip › pharmaceutics-3383215-supplementary.pdf]

Supplementary Figures

A

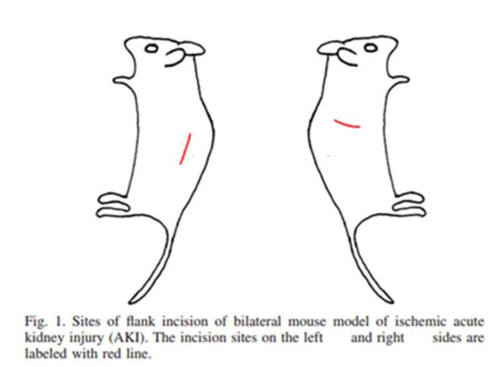

B

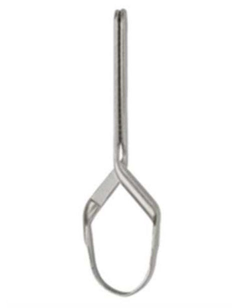

C

Anim. Nu	Ear tag	Body weight	Anesthesia time	Right kidney ischemia	Reperfusion time	Buprenorfin	Warm saline 0.5-1ml	Administered polymer	Woke up	Comments
1										
2										
3										
4										
5										
6										
7										
8										
9										

Figure S1. The incision locations for left and right kidney clamping-adapted from Ref (25) (A) Micro serrefines –clips from Fine Science Tools (FST) (B) Surgery logbook (C).

Table S1. Primer sequences for real-time PCR.

Target gene	Forward primer (5′→3′)	Reverse primer (5′→3′)
RPLP0	TCCAGGCTTTGGGCATCA	CTTTATCAGCTGCACATCACTCAGA
E-selectin	AGCAGAGTTTCACGTTGCAGG	TGGCGCAGATAAGGCTTCA
TNF-α	AGGCTGCCCCGACTACGT	GACTTTCTCCTGGTATGAGATAGCAAA
IL-1β	TCGCTCAGGGTCACAAGAAA	CATCAGAGGCAAGGAGGAAAAC
P-selectin	GCCAGTTCATGTGCGATGAA	GGCGAAGATTCCTGGACACTT
VCAM-1	TGAACCCAAACAGAGGCAGAGT	GGTATCCCATCACTTGAGCAGG
